# CT texture analysis reliability in pulmonary lesions: the influence of 3D vs. 2D lesion segmentation and volume definition by a Hounsfield-unit threshold

**DOI:** 10.1007/s00330-023-09500-8

**Published:** 2023-03-22

**Authors:** Gabriel Adelsmayr, Michael Janisch, Ann-Katrin Kaufmann-Bühler, Magdalena Holter, Emina Talakic, Elmar Janek, Andreas Holzinger, Michael Fuchsjäger, Helmut Schöllnast

**Affiliations:** 1 Division of General Radiology, Department of Radiology, Medical University of Graz, Auenbruggerplatz 9, 8036 Graz, Austria; 2 Institute for Medical Informatics, Statistics and Documentation, Medical University of Graz, Auenbruggerplatz 2/9/V, 8036 Graz, Austria; 3 Institute of Radiology, LKH Graz II, Göstinger Strasse 22, 8020 Graz, Austria

**Keywords:** Humans, Reproducibility of results, Lung, Tomography, X-ray computed, Lung neoplasms

## Abstract

**Objective:**

Reproducibility problems are a known limitation of radiomics. The segmentation of the target lesion plays a critical role in texture analysis variability. This study’s aim was to compare the interobserver reliability of manual 2D vs. 3D lung lesion segmentation with and without pre-definition of the volume using a threshold of − 50 HU.

**Methods:**

Seventy-five patients with histopathologically proven lung lesions (15 patients each with adenocarcinoma, squamous cell carcinoma, small cell lung cancer, carcinoid, and organizing pneumonia) who underwent an unenhanced CT scan of the chest were included. Three radiologists independently segmented each lesion manually in 3D and 2D with and without pre-segmentation volume definition by a HU threshold, and shape parameters and original, Laplacian of Gaussian–filtered, and wavelet-based texture features were derived. To assess interobserver reliability and identify the most robust texture features, intraclass correlation coefficients (ICCs) for different segmentation settings were calculated.

**Results:**

Shape parameters had high reliability (64–79% had excellent and good ICCs). Texture features had weak reliability levels, with the highest ICCs (38% excellent or good) found for original features in 3D segmentation without the use of a HU threshold. A small proportion (4.3–11.5%) of texture features had excellent or good ICC values at all segmentation settings.

**Conclusion:**

Interobserver reliability of texture features from CT scans of a heterogeneous collection of manually segmented lung lesions was low with a small proportion of features demonstrating high reliability independent of the segmentation settings. These results indicate a limited applicability of texture analysis and the need to define robust texture features in patients with lung lesions.

**Key Points:**

*• Our study showed a low reproducibility of texture features when 3 radiologists independently segmented lung lesions in CT images, which highlights a serious limitation of texture analysis.*

*• Interobserver reliability of texture features was low regardless of whether the lesion was segmented in 2D and 3D with or without a HU threshold.*

*• In contrast to texture features, shape parameters showed a high interobserver reliability when lesions were segmented in 2D vs. 3D with and without a HU threshold of − 50.*

**Supplementary Information:**

The online version contains supplementary material available at 10.1007/s00330-023-09500-8.

## Introduction

Radiomics, which involves the extraction of a large number of quantitative features from radiology images, may provide additional information on the lesion composition [1], can potentially predict survival in patients with colorectal cancer, high-grade gliomas, and renal cell carcinomas [2–4], and has prognostic power in head-and-neck cancer [5]. In the lung, radiomics has the potential to differentiate between benign and malignant lung nodules [6], to predict the prognosis for non–small cell lung cancer [5, 7], and to distinguish between different types of lung cancer [7]. Nevertheless, to obtain a final diagnosis, patients in a clinical setting routinely undergo percutaneous or transbronchial tissue sampling, which carries the risk of severe complications [8–11]. Concerns about the reproducibility of radiomics, especially texture features, constitute one of the main obstacles to the clinical acceptance and implementation of CT texture analysis (CTTA) [12]. The segmentation of the lesion of interest is considered a substantial source of inadequate reproducibility [13]. Manual segmentation is the reference standard in most trials when it is performed by experts; however, lesion delineation is prone to variability. Whether the lung lesion should be segmented in three dimensions (3D) or only in the maximum diameter in two dimensions (2D) remains controversial. The application of a Hounsfield-unit (HU) threshold in order to restrict the volume before segmentation of pulmonary lesions may also influence CTTA results. A − 50 HU threshold for CTTA in the lung has been proposed as a means to prevent the potential segmentation of extra-lesional pulmonary tissue [14, 15]. However, sub-solid parts of lung lesions, as previously reported characteristic of adenocarcinomas [16], may also be excluded when using such a threshold. Since reliable reproducibility is a pre-requisite for the applicability of CTTA and the optimum segmentation settings for pulmonary lesions remain controversial, the aim of our study was to compare the interobserver reliability of CTTA parameters derived with manual 2D vs. 3D lung lesion segmentation, with and without pre-definition of the volume using a threshold of − 50 HU.

## Materials and methods

### Patient population

The Institutional Ethics Committee approved this retrospective, single-institution study.

A search in the institution’s data system yielded 1049 patients who underwent CT-guided biopsy of the lung at our institution between January 2013 and December 2018 for whom both a pre-interventional planning CT examination without contrast administration and a corresponding histopathologic diagnosis were available. To represent the heterogeneity of suspicious lung lesions encountered in routine clinical thoracic imaging, we selected patients with the histopathologic diagnosis of adenocarcinoma, squamous cell carcinoma, carcinoid, small cell lung cancer, or organizing pneumonia. Overall, 75 patients (47 males, 28 females) were included. Because our data search retrieved only 15 patients with the diagnosis of carcinoid, we included 15 consecutive patients each with adenocarcinoma, squamous cell carcinoma, small cell lung cancer, and organizing pneumonia in chronological order starting with the earliest to maintain homogeneity of group size.

### CT imaging

All CT-guided biopsies were performed on the same 128-multislice-CT scanner (Somatom Definition AS, Siemens Healthineers). A CT scan without contrast (craniocaudal scanning direction; tube voltage: 100 kV; tube current: 100 mAs; collimation: 128 × 0.6 mm; pitch: 1.2; section thickness: 3 mm; increment: 3.0 mm; reconstruction: standard kernel using iterative reconstruction) was carried out to determine the exact position of the target lesion and to plan the access route for the biopsy needle for each patient. These pre-procedure CT scans were used for CTTA of all lesions.

### CT texture analysis (CTTA)

Image segmentation was performed with the open-source 3D Slicer software (version 4.11.0–2019-03–24), and radiomic feature extraction was done with SlicerRadiomics, a freeware extension to 3D Slicer. CT images were extracted from the institution’s PACS in DICOM format and transferred to 3D Slicer. Lesion segmentations were performed manually and independently by three radiologists (G.A., M.J., and A.K., with 4–8 years of experience in chest CT image interpretation), who were blinded to the histopathologic diagnosis.

For lesion segmentation, the borders of the target lung nodules and masses were manually drawn in the CT lung window setting (center, − 600 HU; width, 1200 HU) in pre-interventional, non-contrast-enhanced CT images. Lesions were segmented in 3D slice by slice in axial slice orientation and in 2D in the largest diameter in axial slice orientation, including perilesional ground-glass opacities when present. Bones and soft tissue not considered part of the target lesion were excluded. Additional analyses of the 3D and 2D segmentations were performed with an attenuation threshold that excluded density values below − 50 HU to avoid accidentally segmenting air, as previously described elsewhere [17].

Texture analysis included first-order features (18 features describing gray-level values), gray-level co-occurrence matrix (GLCM) features (24 features relating the frequencies of pairs of pixels or voxels with certain values and a specified spatial relationship), gray-level dependence matrix (GLDM) features (14 features based on the number of connected pixels or voxels within a distance dependent on the center), gray-level run length matrix (GLRLM) features (16 features based on the length in number of consecutive pixels or voxels having the same gray-level value), gray-level size zone matrix (GLSZM) features (16 features, which quantify gray-level zones defined by the number of pixels or voxels with the same gray-level value), and neighboring gray tone difference matrix (NGTDM) features (5 features describing the difference between a gray-level value and the mean of its neighbors within a certain distance) [12]. Additionally 14 parameters representing shape characteristics of the segmented lesion were analyzed.

A Laplacian of Gaussian (LoG) filter was applied to produce fine-to-coarse texture features with filter values of 0.5, 1.5, and 2.5, as previously described in CTTA of the lung [14, 17, 18].

In addition, 744 wavelet-based texture features with four different band combinations (low-low, high-high, high-low, low–high) were derived.

Overall, 1130 radiomic features were included in the analysis.

### Statistical analysis

Descriptive statistics are presented as absolute and relative frequencies for categorical data and as means and standard deviations (SD) or medians and ranges for continuous data. Agreement in the segmented lesions between different raters was evaluated by an intraclass correlation coefficient (ICC) for each of the 1130 radiomic features separately. The ICC was assessed by a two-way random model (ICC (2,1) concept). ICC values were defined as poor (< 0.50), moderate (0.50–0.75), good (0.75–0.90), or excellent (> 0.90), according to Koo and Li [19]. The statistical analyses were performed with the SAS software (version 9.4; SAS Institute, Inc.).

## Results

Detailed information on patients’ demographics and the segmented lung lesions is provided in Table 1. Two patients with organizing pneumonia and three patients with carcinoids were excluded from the analysis when applying a threshold of − 50 HU, because there remained no computable lesion above this density.

**Table 1:** Patients’ demographics, histopathological tumor grading and tumor size

Entity*	Number of patients	Median age (range)	Gender (f)	Tumor size^#^	Histopathologic grading				GGO^~^ present
					G1	G2	G3		
AC^+^	15	70 (45–89) years	7 (46.7%)	41 ± 20 mm (range, 18–96 mm)		7	6	4/15	
SQCC	15	70 (45–78) years	2 (13.3%)	51 ± 33 mm (range, 14–139 mm)	2	3	10	1/15	
SCLC	15	65 (55–81) years	5 (33.3%)	52 ± 36 mm (range, 17–133 mm)				1/15	
Carcinoid	15	69 (54–78) years	10 (66.7%)	25 ± 6 mm (range, 16–40 mm)	14	1		0/15	
OP	15	65 (38–84) years	4 (26.7%)	47 ± 25 mm (range, 16–92 mm)				8/15	

*f* female

^*^AC, adenocarcinoma; *SQCC*, squamous cell carcinoma; *SCLC*, small cell lung carcinoma; *OP*, organizing pneumonia

^#^Mean largest tumor size (± standard deviation) and range in three dimensions

^+^No histopathologic grading was available in 2 patients with AC

^~^Ground-glass opacities

**Fig. 1:**
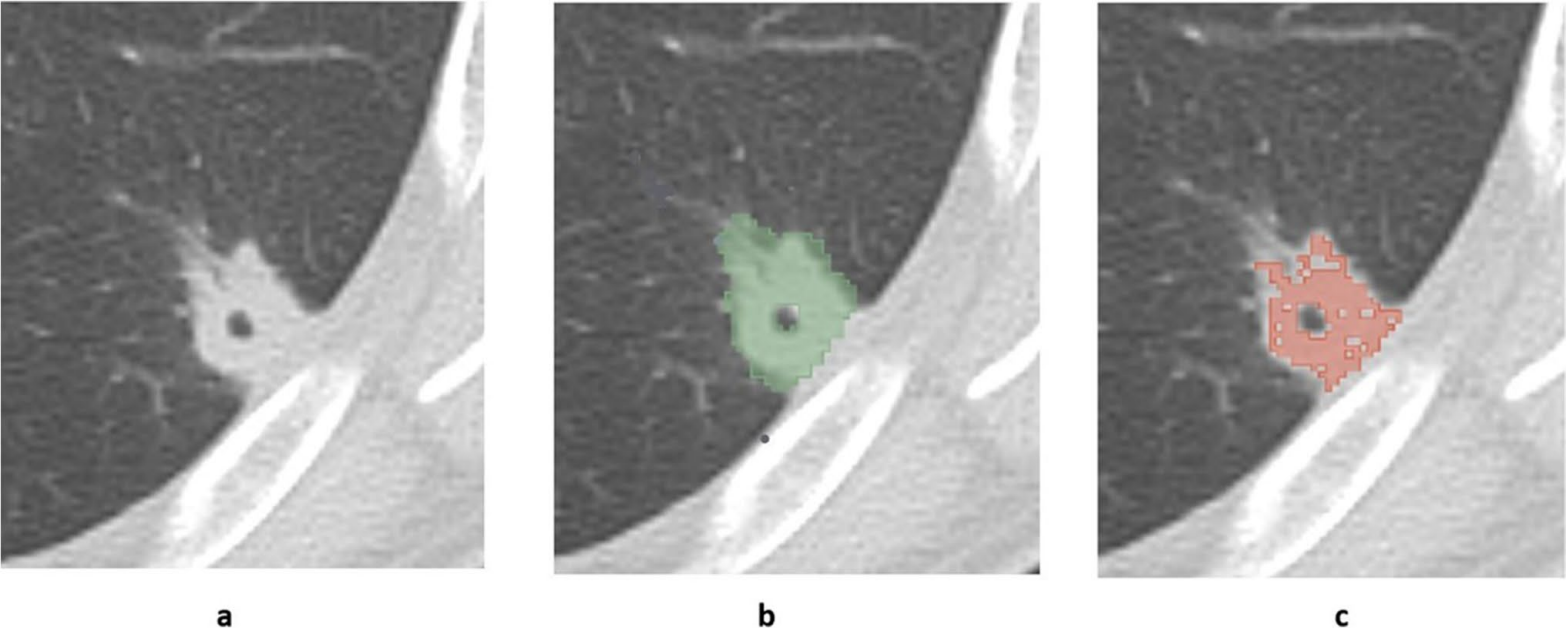
**a–c** Non-contrast-enhanced CT image of a 50-year-old male patient with histologically proven organizing pneumonia without segmentation (**a**), with green-colored segmentation without a HU threshold (**b**) and with red-colored segmentation with a threshold of − 50 HU (**c**). Example is shown in maximum diameter only

**Fig. 2:**
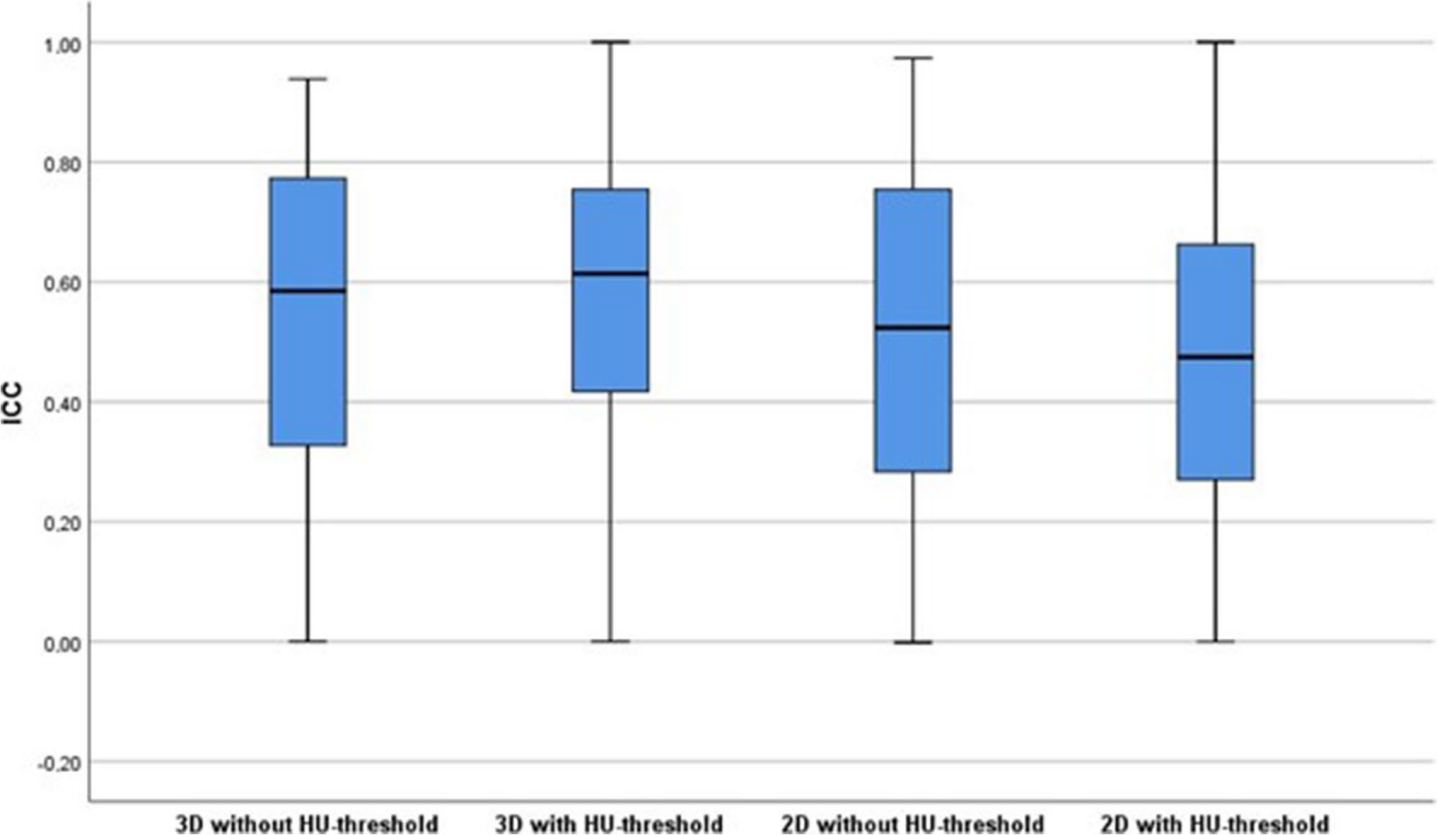
Boxplots including all radiomics features (shape, original, LoG-filtered, wavelet-based) for 3D and 2D segmentations without and with application of a HU threshold of − 50

Figure 1 shows an example of a lung lesion from a CT scan of a patient with organizing pneumonia segmented with and without a density threshold of − 50 HU in maximum axial diameter. Additional figures demonstrating segmentations of adenocarcinoma, squamous cell carcinoma, carcinoid, and small cell lung cancer are provided as electronic supplementary material. The ICCs of all 1130 extracted radiomic features in 3D and 2D segmentation without and with a density threshold of − 50 HU were distributed over a wide range (Fig. 2).

Excellent or good ICC values at all four different segmentation settings were found for 8 (57.1%) shape parameters, 4 (4.3%) original features, 32 (11.5%) LoG-filtered texture features, and 50 (6.7%) wavelet-based texture features (Table 2).

**Table 2:** Texture features with excellent or good ICC values for all 4 segmentation settings (limited to the 5 features with the highest ICC values)

Original features (ICC range)	LoG-filtered features (ICC range)	Wavelet-based features (ICC range)
Original_glrlm_GrayLevelNonUniformity (0.89–0.94)	log-sigma-1.5_gldm_GrayLevelNonUniformity (0.89–0.96)	HHH_gldm_DependenceNonUniformity (0.88–0.97)
Original_glszm_GrayLevelNonUniformity (0.78–0.97)	log-sigma-2.5_gldm_GrayLevelNonUniformity (0.90–0.95)	HHH_firstorder_TotalEnergy (0.89–0.96)
Original_gldm_GrayLevelNonUniformity (0.83–0.96)	log-sigma-2.5_gldm_DependenceNonUniformity (0.87–0.95)	HLH_gldm_DependenceNonUniformity (0.88–0.97)
	log-sigma-1.5_gldm_DependenceNonUniformity (0.88–0.95)	HLL_gldm_DependenceNonUniformity (0.88–0.96)
	log-sigma-0.5_gldm_DependenceNonUniformity (0.89–0.96)	LHH_gldm_DependenceNonUniformity (0.88–0.96)

**Fig. 3:**
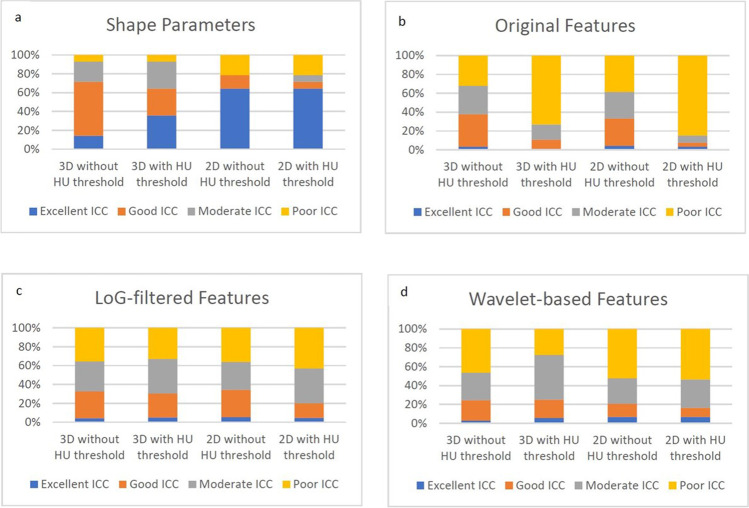
**a–d** ICC values categorized into poor (< 0.50), moderate (0.50–0.75), good (0.76–0.90), and excellent (> 0.90) for segmentations in 3D and 2D with and without a density threshold for shape parameters (**a**) and original (**b**), LoG-filtered (**c**), and wavelet-based (**d**) features, displayed in percentages

The majority (8/14) of shape parameters had excellent or good ICC values at all segmentation settings. ICC values were highest for 2D segmentations without a HU threshold (79% excellent or good). For shape parameters derived after 2D segmentation with a HU threshold as well as those derived after 3D segmentation without a HU threshold, 71% of ICC values were excellent or good. For shape parameters segmented in 3D with a HU threshold, 64% of ICC values were excellent or good. Details of interobserver reliability for shape parameters are shown in Table 3 and Fig. 3.

**Table 3:** Interobserver reliability of shape parameters and original, LoG-filtered and wavelet-based features

	**Excellent ICC** **No. (%)**	**Good ICC** **No. (%)**	**Moderate ICC** **No. (%)**	**Poor ICC** **No. (%)**
Shape parameters				
3D without HU threshold	2 (14.3%)	8 (57.1%)	3 (21.4%)	1 (7.1%)
3D with HU threshold	5 (35.7%)	4 (28.6%)	4 (28.6%)	1 (7.1%)
2D without HU threshold	9 (64.2%)	2 (14.3%)	0 (0%)	3 (21.4%)
2D with HU threshold	9 (64.2%)	1 (7.1%)	1 (7.1%)	3 (21.4%)
Original features				
3D without HU threshold	3 (3.2%)	32 (34.4%)	28 (30.1%)	30 (32.3%)
3D with HU threshold	1 (1.1%)	9 (9.7%)	15 (16.1%)	68 (73.1%)
2D without HU threshold	4 (4.3%)	27 (29%)	26 (30.0%)	36 (38.7%)
2D with HU threshold	3 (3.2%)	4 (4.3%)	7 (7.5%)	79 (84.9%)
LoG-filtered features				
3D without HU threshold	12 (4.3%)	79 (28.3%)	89 (31.9%)	99 (35.5%)
3D with HU threshold	14 (5.0%)	71 (25.%)	102 (36.6%)	92 (33.0%)
2D without HU threshold	15 (5.4%)	81 (29.0%)	82 (29.4%)	101 (36.2%)
2D with HU threshold	13 (4.7%)	43 (15.4%)	103 (36.9%)	120 (43.0%)
Wavelet-based features				
3D without HU threshold	21 (2.8%)	162 (21.8%)	217 (29.2%)	344 (46.2%)
3D with HU threshold	41 (5.5%)	145 (19.5%)	353 (47.5%)	205 (27.6%)
2D without HU threshold	50 (6.7%)	103 (13.8%)	202 (27.2%)	389 (52.3%)
2D with HU threshold	49 (6.6%)	73 (9.8%)	223 (30.0%)	399 (53.6)

ICCs of 93 original features (first order, GLCM, GLDM, GLRLM, GLSZM, NGTDM) were calculated in 2D and 3D with and without a HU threshold of − 50. Higher ICC values were observed when segmentation was performed without a HU threshold, both for 3D (38% excellent or good) and 2D segmentation (33% excellent or good), as opposed to with a HU threshold. Original features derived from segmentations performed with a HU threshold had remarkably low ICC values (3D: 11% excellent or good; 2D: 8% excellent or good; Table 3 and Fig. 3).

LoG filters of 0.5, 1.5, and 2.5 were applied on original features, resulting in a total of 279 LoG-filtered parameters that were analyzed for interobserver reliability. ICC values for these parameters were higher when segmentations were performed without a HU threshold as opposed to with a HU threshold of − 50 for both 3D segmentation (33% excellent or good vs. 31% excellenct or good) and 2D segmentation (34% excellent or good vs. 20% excellent or good).

Details regarding ICCs for original and LoG-filtered features are presented in Table 3 and Fig. 3.

A total of 744 wavelet-based texture parameters with four different band combinations were derived. Among wavelet-based texture parameters derived from 3D segmentations, ICC values did not differ substantially between parameters derived after application of a HU threshold (25.0% excellent or good) and those derived without a HU threshold (24.6% excellent or good). For wavelet-based texture parameters derived from 2D segmentations, however, the application of a HU threshold yielded even lower ICC values (16.4% excellent or good) than did omission of the HU threshold (20.6% excellent or good). Detailed results on the interobserver reliability of wavelet-based texture parameters are given in Table 3.

## Discussion

This study explored the influence of 2D vs. 3D manual segmentation with and without the application of a HU threshold of − 50 on the reliability of CTTA in different types of histologically proven pulmonary lesions. Overall, the reliability of texture analysis for original, LoG-filtered, and wavelet-based texture features was rather low, with the percentages of these features demonstrating excellent or good ICCs ranging from just 8 to 38%. The highest proportion of excellent and good ICC values was found when segmentation was performed in 3D without a HU threshold, whereas the lowest proportion of excellent and good ICC values was observed for 2D segmentation with a HU threshold.

Radiomics is a promising approach for non-invasive tissue characterization of lesions based on image analysis [1]. Several studies, however, have identified the lesion segmentation process as a relevant source of variability of texture analysis [20]. Tumor type and tumor site have been shown to influence interobserver variability in lesion delineation and therefore radiomics analysis [21]. In renal masses, investigation of intra- and interobserver variability showed greater reliability of segmentation in contrast-enhanced CT vs. non-enhanced CT [12], and texture analysis differed by the phase of the contrast injection protocol [22]. For PET/CT in non–small cell lung cancer (NSCLC), radiomic features had high test–retest and interobserver stability [23] similar to the more commonly used PET parameters SUV_max_, SUV_mean_, and SUV_peak_, and the performance of radiomic features depended more on the delineation method than on the applied reconstruction method [24].

Studies on the reliability of CTTA of pulmonary lesions in different segmentation settings are scarce. In patients with NSCLC, feature reliability varied between manual and different semi-automatic segmentation approaches [25]. Representations of pulmonary tumor heterogeneity and reproducibility in 3D segmentation were previously described as superior compared to those in 2D segmentation [26, 27]. Maximum-diameter-only 2D segmentations could provide the benefit of reducing potential motion and breathing artifacts that might be more pronounced when more CT slices are segmented in three dimensions. On the other hand, 3D segmentation includes the whole tumor and may better depict the average tissue properties for lesion classification in CTTA. A potential drawback of 3D segmentation, however, is that a higher number of segmented image slices could lead to inclusion of a larger quantity of potentially non-tumorous tissue that may distort the results. In the present study, the largest differences in ICC values between 3D and 2D segmentations were found for LoG-filtered texture features derived after use of a HU threshold (3D: 31% excellent or good vs. 2D: 20% excellent or good ICC values) and for wavelet-based parameters derived after pre-segmentation use of a HU threshold (3D: 25.0% excellent or good vs. 2D: 16.4% excellent or good ICC values). For all other combinations of segmentation settings, the differences in ICC values between 3D and 2D were smaller, but nevertheless, 3D segmentations always resulted in higher ICCs than did 2D segmentations.

The most pronounced differences in ICC values were observed between original texture features derived after 3D or 2D segmentation without a HU threshold (38% and 33% excellent or good ICC values, respectively) and those derived after 3D or 2D segmentation with a HU threshold of − 50 (11% and 8% excellent or good ICC values, respectively). Except for wavelet-based features derived from 3D segmentation, which showed minimally higher ICC values with than without application of a HU threshold (25% excellent or good vs. 24.6% excellent or good), all other parameters derived from 3D and 2D segmentations showed a higher proportion of excellent and good ICC values when segmentation was performed without a HU threshold. Although it has been postulated that applying a − 50 HU threshold for CTTA could prevent the potential segmentation of extra-lesional pulmonary tissue [14, 15], it remains unclear whether a HU threshold excludes not only negligible extra-lesional parenchymal changes but also characteristic sub-solid parts of lung lesions, as previously reported for adenocarcinomas [16]. Moreover, the optimum value of the HU threshold that should be applied remains uncertain. In our study cohort, where one fifth of lung lesions (14 out of 75, Table 1) had associated ground-glass opacities, the results favor segmentation without a HU threshold, especially when original texture features are to be analyzed. Notably the overall reliability of texture features was lower than previously reported [12, 21]. This could be due to the selection of lung lesions in our study. In order to represent a realistic scenario encountered in clinical practice, we included various types of lung lesions with heterogeneous appearances. This could have led to a substantial variability of tumor delineation from atelectatic lung tissue, large vessels, and mediastinal structures. When using a − 50 HU threshold, two patients with organizing pneumonia and three patients with carcinoids were excluded from our analysis, because there was no computable lesion with a density above this threshold—an indication of the limitations of using a negative density threshold for the segmentation of sub-solid pulmonary lesions.

Shape parameters had excellent-to-good ICCs in over half of all cases. The highest ICCs for shape parameters were found with 2D segmentation without a HU threshold, the lowest with 3D segmentation with a HU threshold. These results suggest a robust interobserver agreement of lesion delineation for 2D segmentations. An explanation for the lower ICCs in 3D segmentation could be that interobserver variation in lesion delineation increases in 3D due to the higher number of slices on which such delineation must be performed.

We were able to identify a small proportion of texture features (Table 2) that had very high ICC values independent of the segmentation settings. Further investigations may be warranted to determine whether these robust texture features can reliably offer support for the characterization of the lesion of interest.

The foremost limitation of the present study was the small number of patients included. Nevertheless, this is the largest study to date investigating the influence of different segmentation settings on the reliability of radiomics of various lung lesions in a clinical setting.

It was previously shown that many radiomic features are non-reproducible and redundant with different CT acquisition parameters and scanners [28]. Therefore, the present study included only patients who were scanned with the same acquisition parameters and on the same CT scanner. Consequently the results of our trial cannot be generalized for different CT scanner types and image acquisition parameters. To test the robustness of radiomics and advance progress toward routine applicability of CTTA, it would have been helpful to be able to assess the influence of different scanner types and image acquisition parameters in our study.

In conclusion, our study demonstrated low interobserver reliability of CT-derived texture features in histopathologically heterogeneous pulmonary lesions, with overall higher reliability when segmentations were performed in 3D without the use of a HU threshold. A small proportion of texture features with very high interobserver reliability independent of segmentation settings were identified. These results indicate a limited applicability of CTTA and the need to define robust texture features for the characterization of various types of pulmonary lesions.

## Supplementary Information

Below is the link to the electronic supplementary material.

Supplementary file1 (PDF 1083 KB)

## Abbreviations

AC: Adenocarcinomas
CTTA: Computed tomography texture analysis
ICC: Intraclass correlation coefficient
LoG: Laplacian of Gaussian
OP: Organizing pneumonia
SCLC: Small cell lung cancers
SQCC: Squamous cell carcinomas

## Declarations

### Guarantor

The scientific guarantor of this publication is Prof. Dr. Michael Fuchsjaeger.

### Statistics and biometry

One of the authors has significant statistical expertise.

### Methodology

• Retrospective.

• Performed at one institution.
