# Supplementary material for: CT texture analysis reliability in pulmonary lesions: the influence of 3D vs. 2D lesion segmentation and volume definition by a Hounsfield-unit threshold
Source: Eur Radiol. 2023 Mar 22;33(5):3064–71. doi: 10.1007/s00330-023-09500-8 (PMC10121537; doi:10.1007/s00330-023-09500-8)

Supplemental Figure 1. 3D segmentation of a pulmonary adenocarcinoma in a male 73 years old patient by three radiologists (a-c) in maximum axial diameter (left) and maximum sagittal diameter (right) without a HU-threshold (green boundary) and with a -50 HU threshold (red boundary).

a.

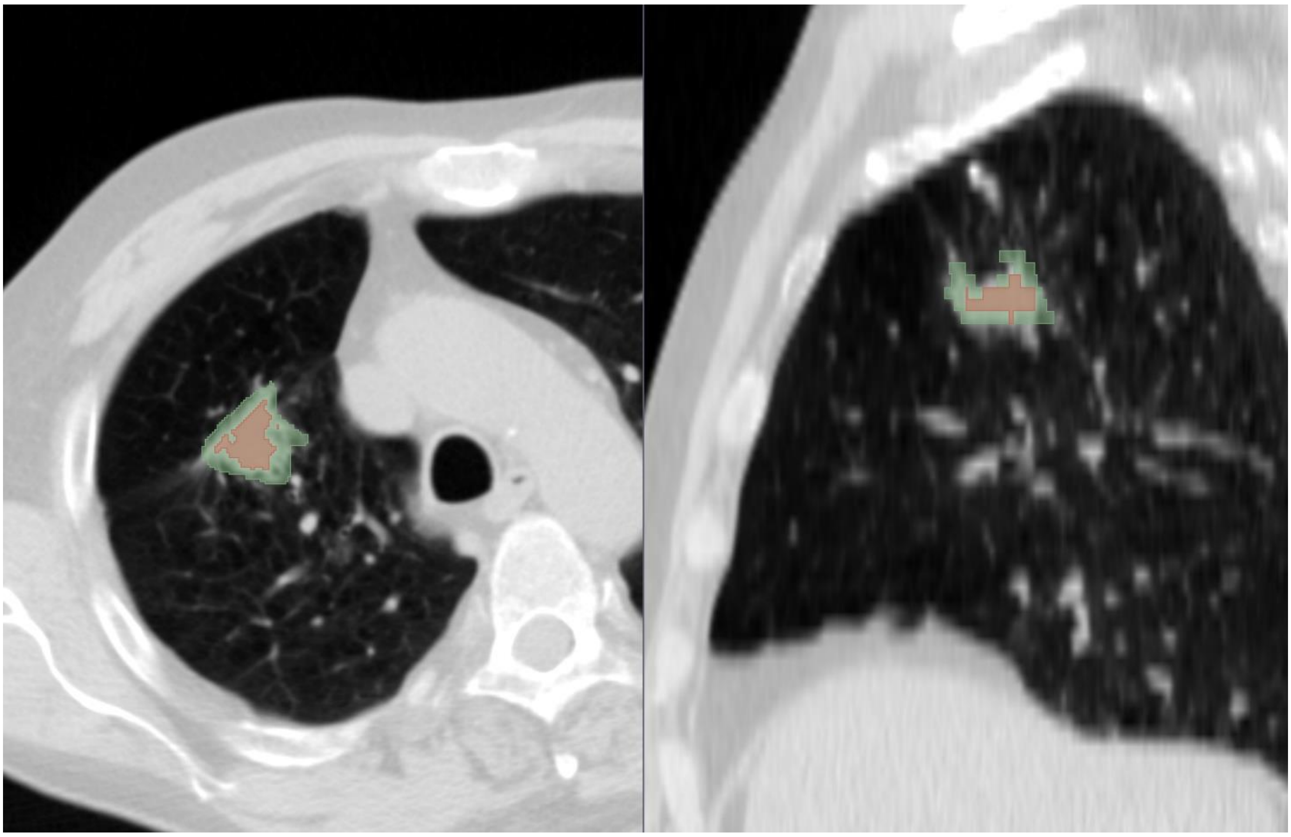

b.

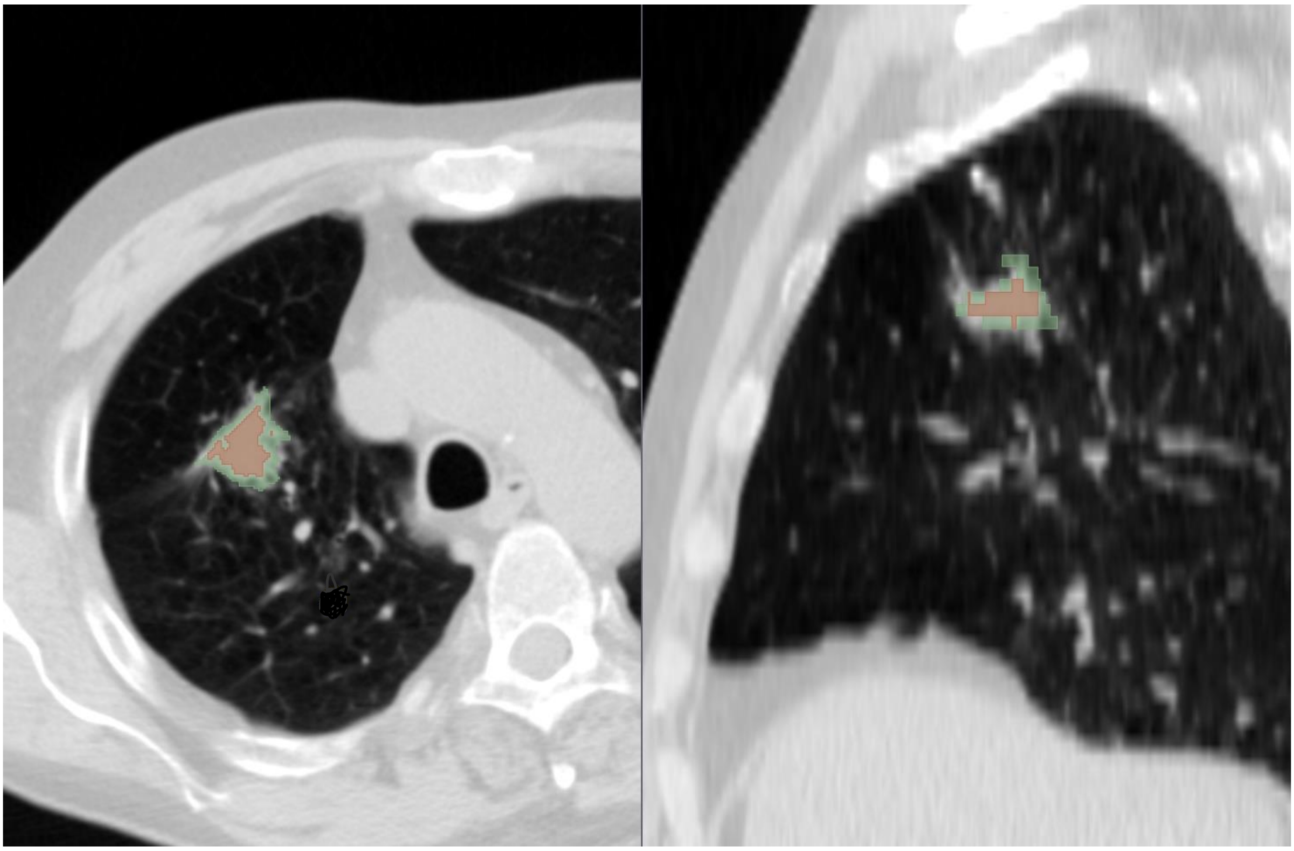

c.

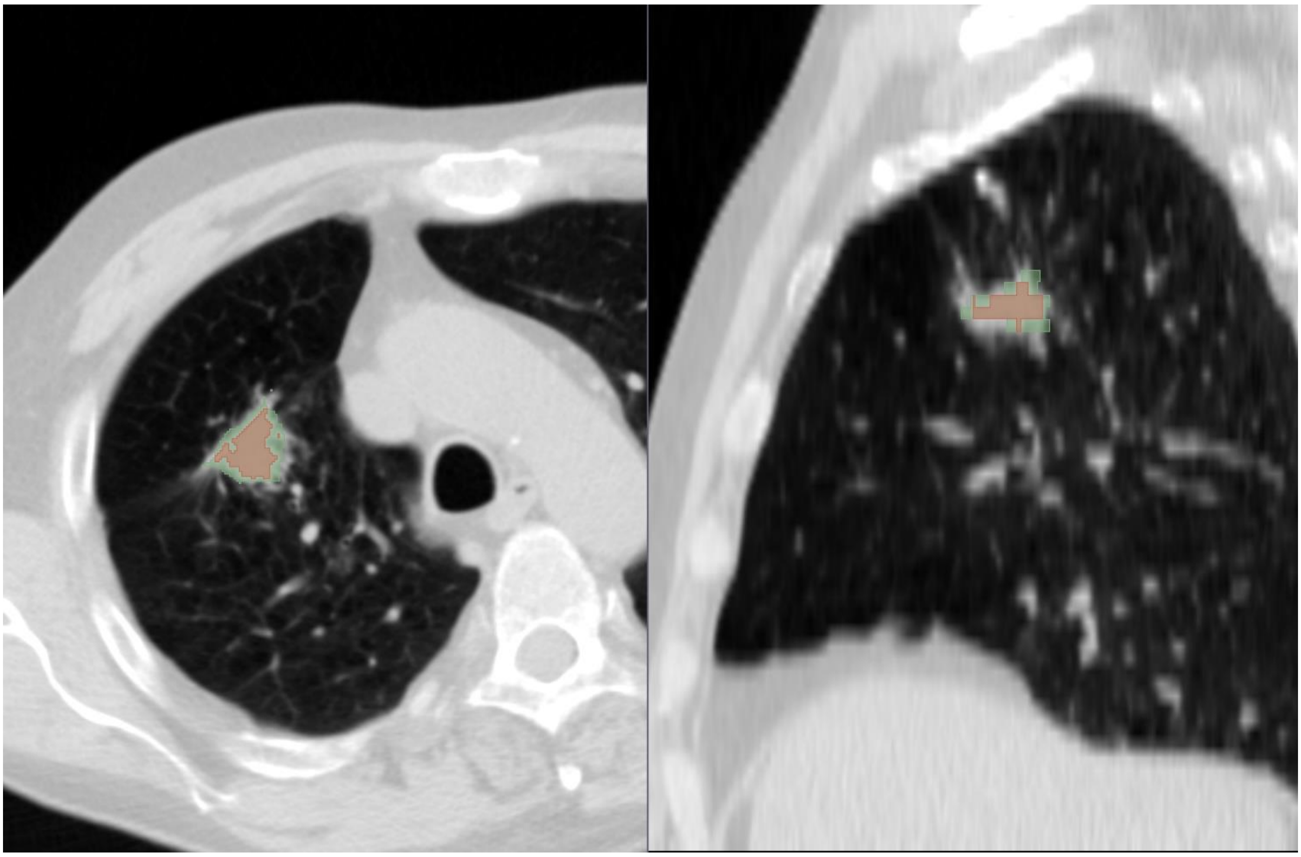

Supplemental Figure 2. 3D segmentation of a pulmonary squamous cell carcinoma in a male 65 years old patient by three radiologists (a-c) in maximum axial diameter (left) and maximum sagittal diameter (right) without a HU-threshold (green boundary) and with a -50 HU threshold (red boundary).

a.

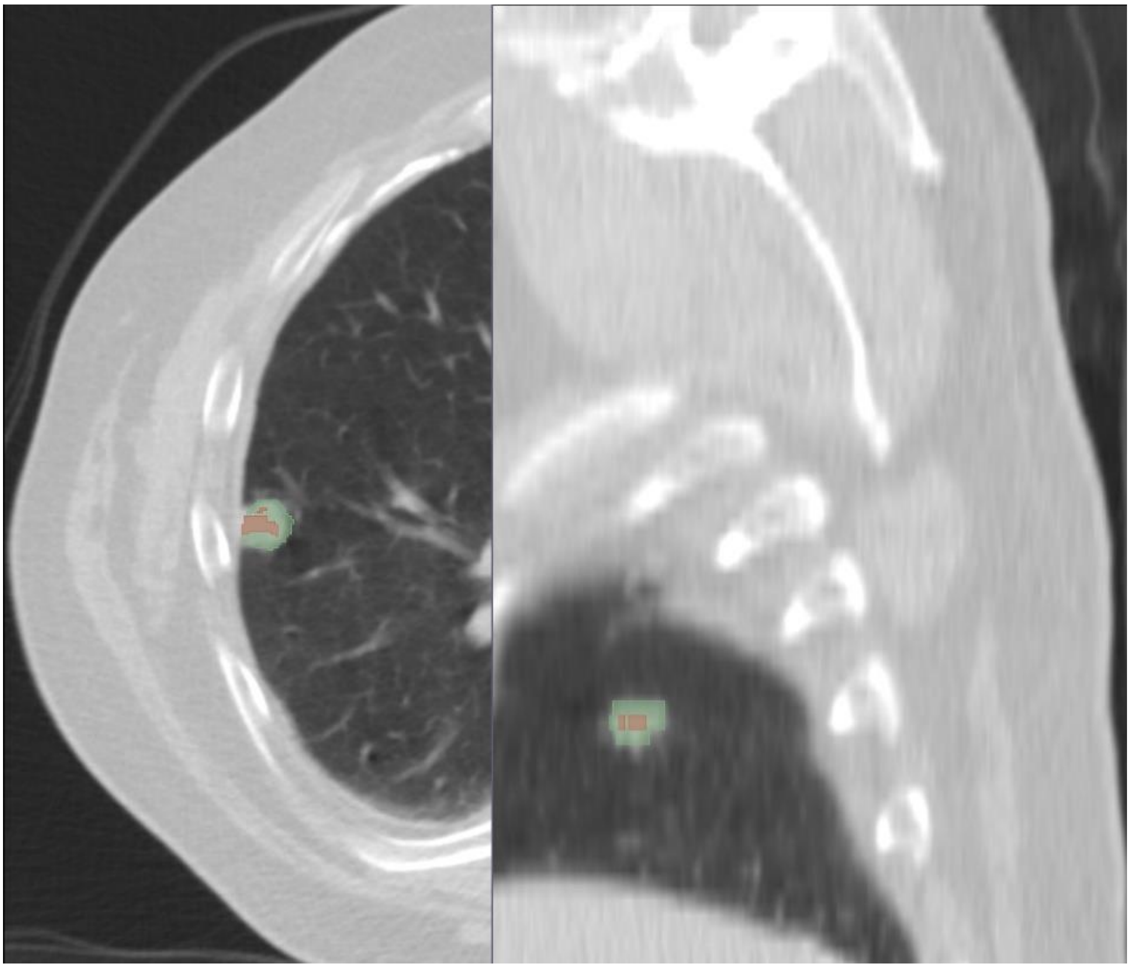

b.

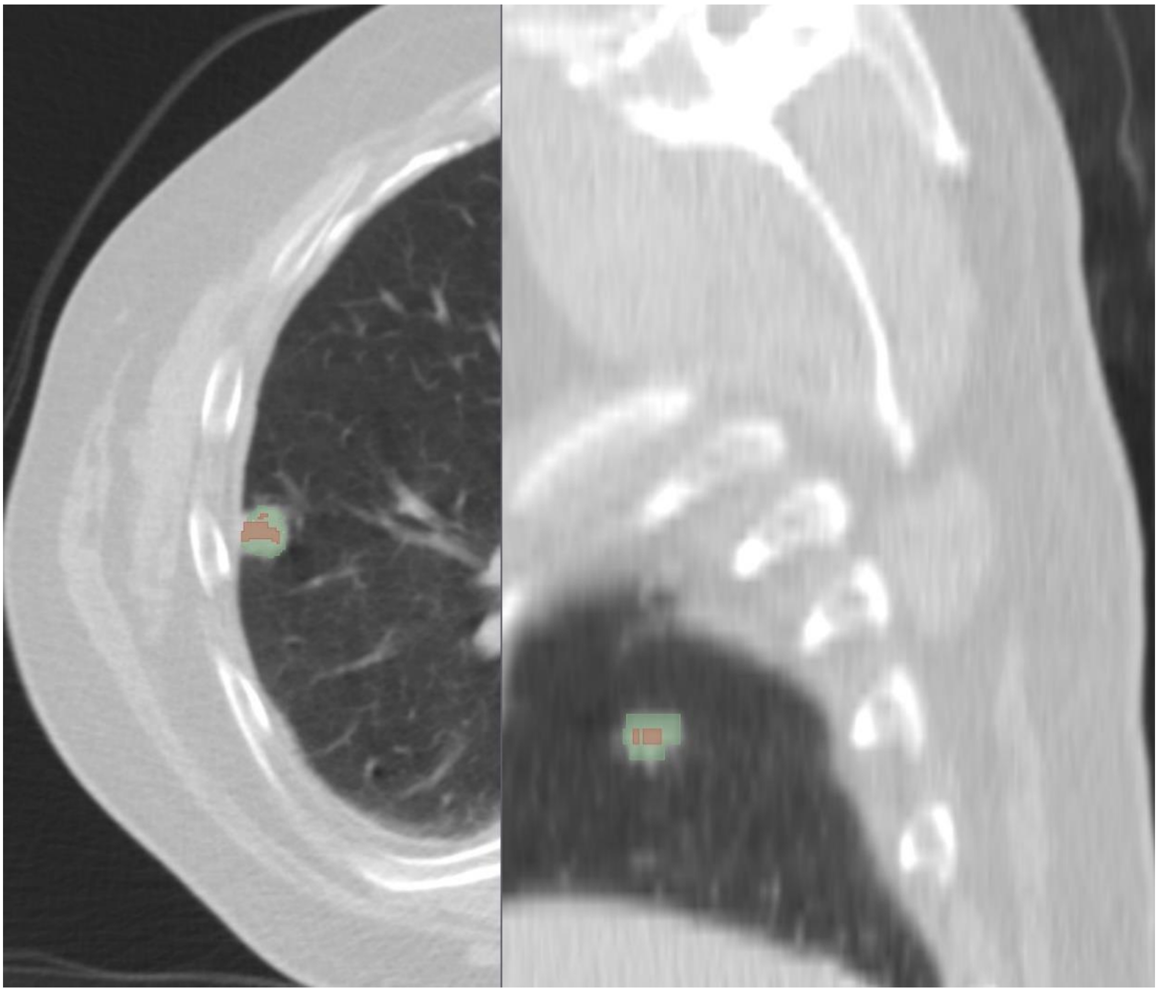

c.

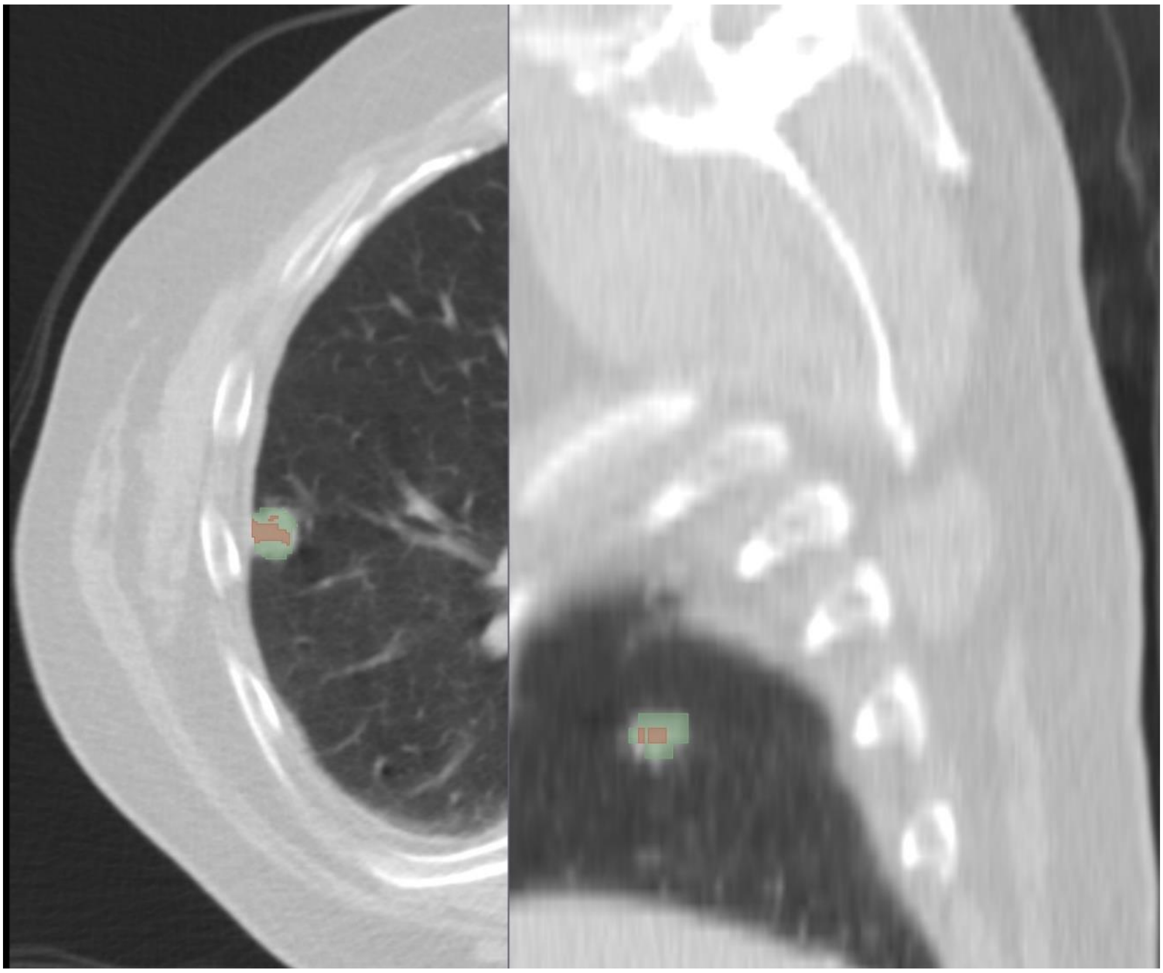

Supplemental Figure 3. 3D segmentation of a pulmonary carcinoid in a female 57 years old patient by three radiologists (a-c) in maximum axial diameter (left) and maximum sagittal diameter (right) without a HU-threshold (green boundary) and with a -50 HU threshold (red boundary).

a.

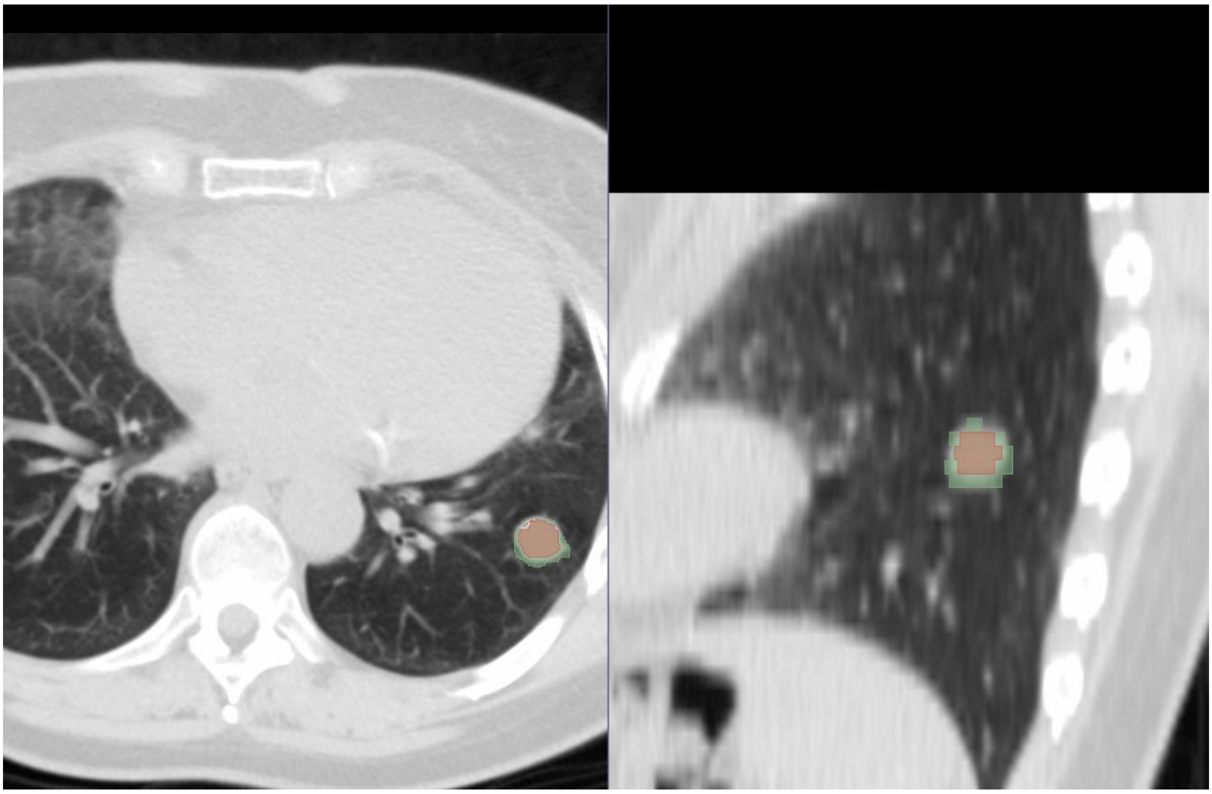

b.

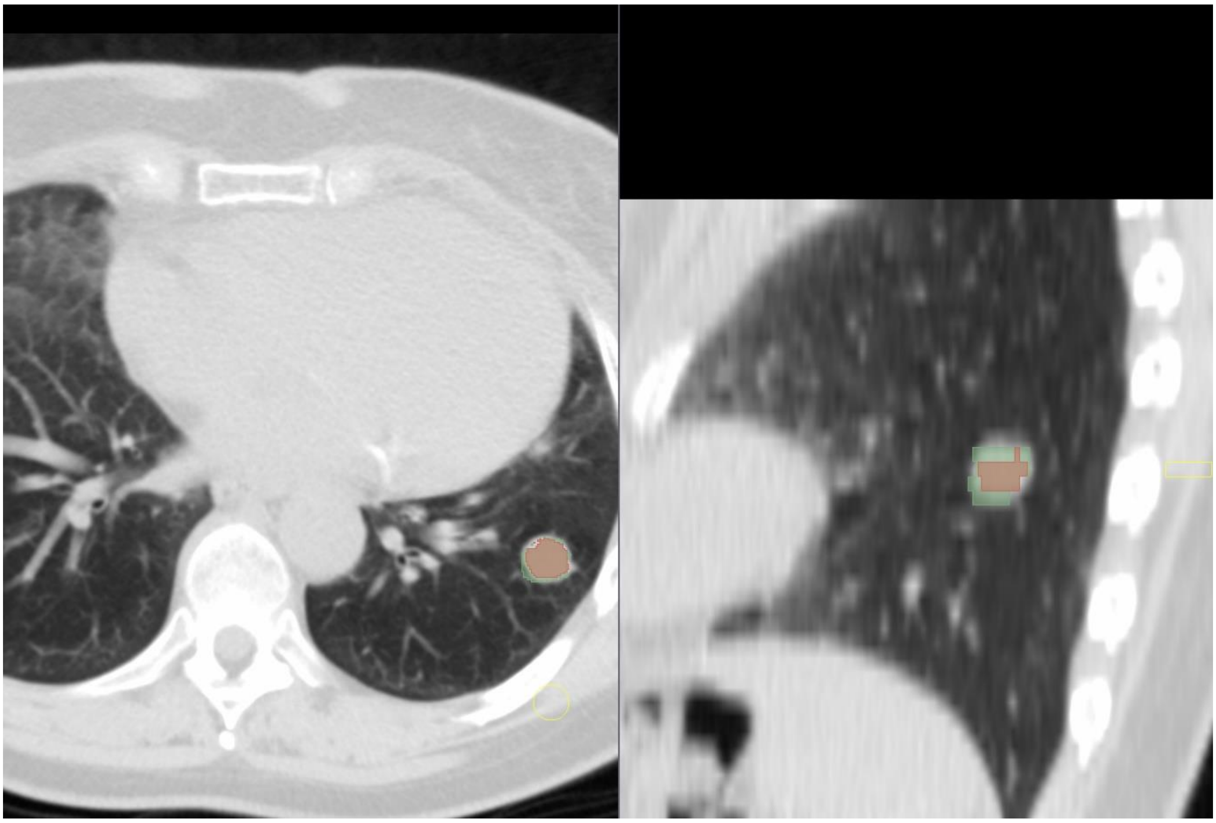

c.

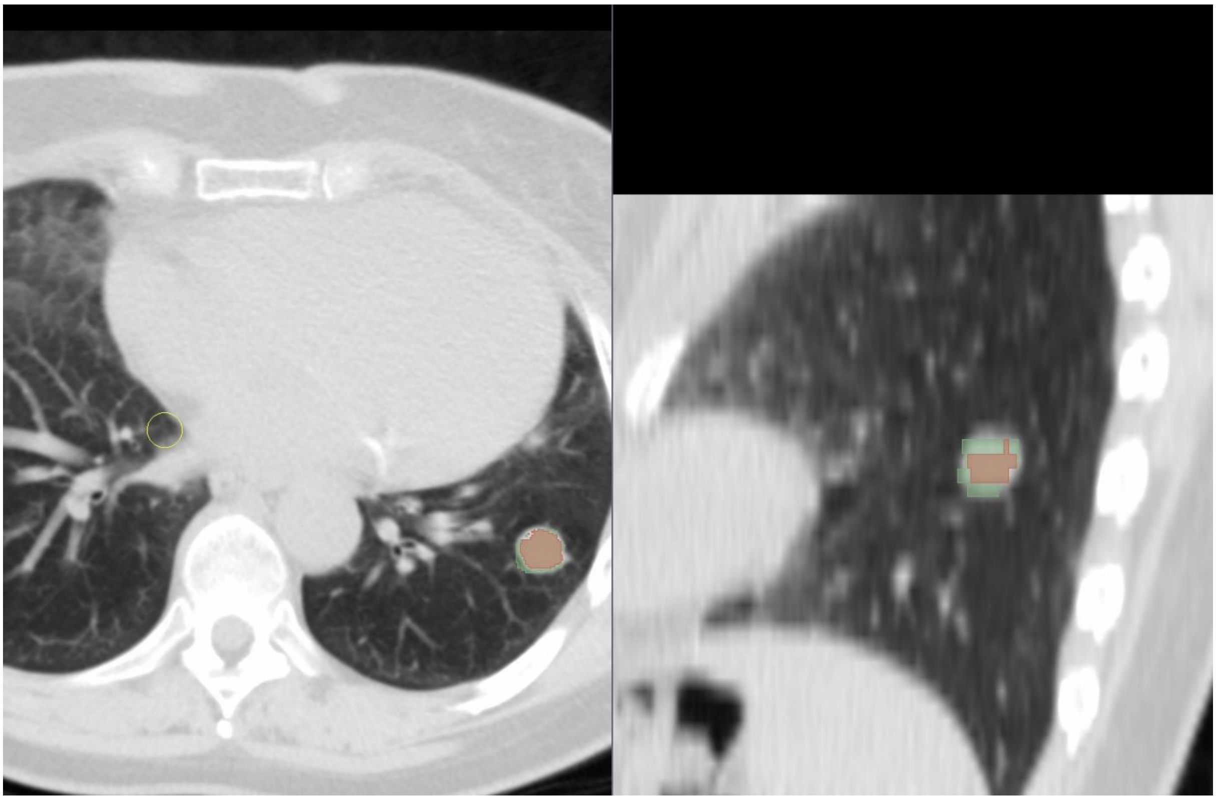

Supplemental Figure 4. 3D segmentation of a small cell lung cancer in a female 67 years old patient by three radiologists (a-c) in maximum axial diameter (left) and maximum sagittal diameter (right) without a HU-threshold (green boundary) and with a -50 HU threshold (red boundary).

a.

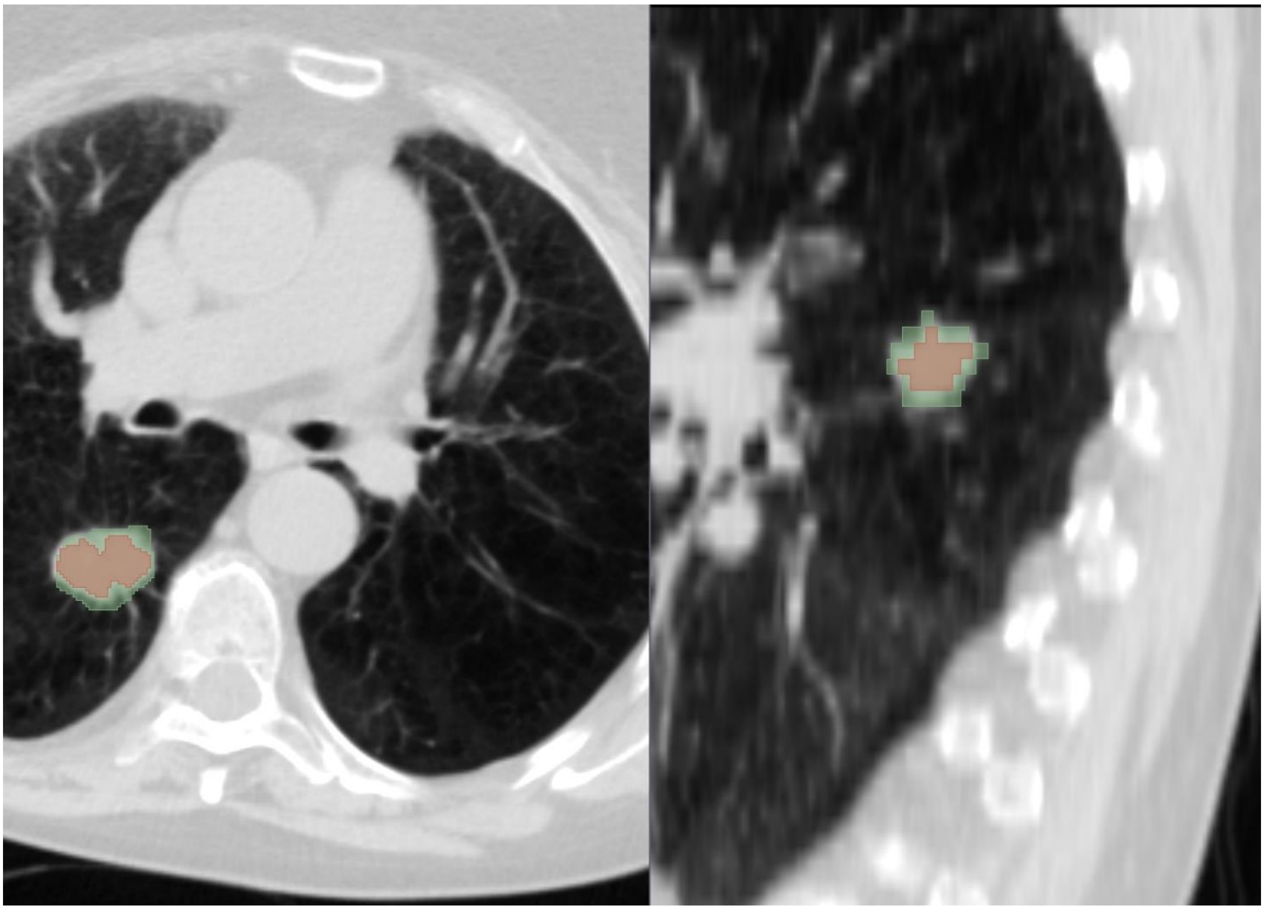

b.

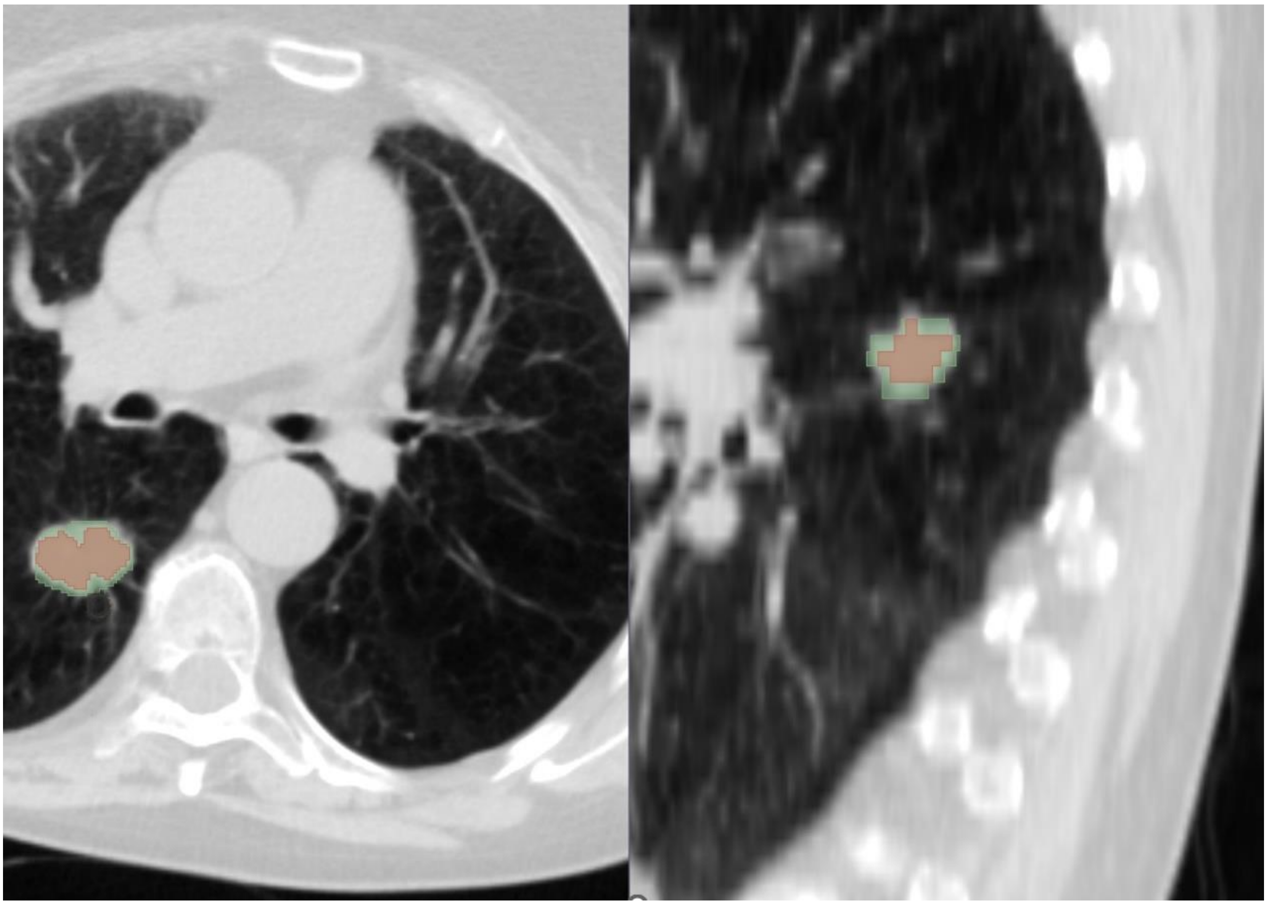

c.

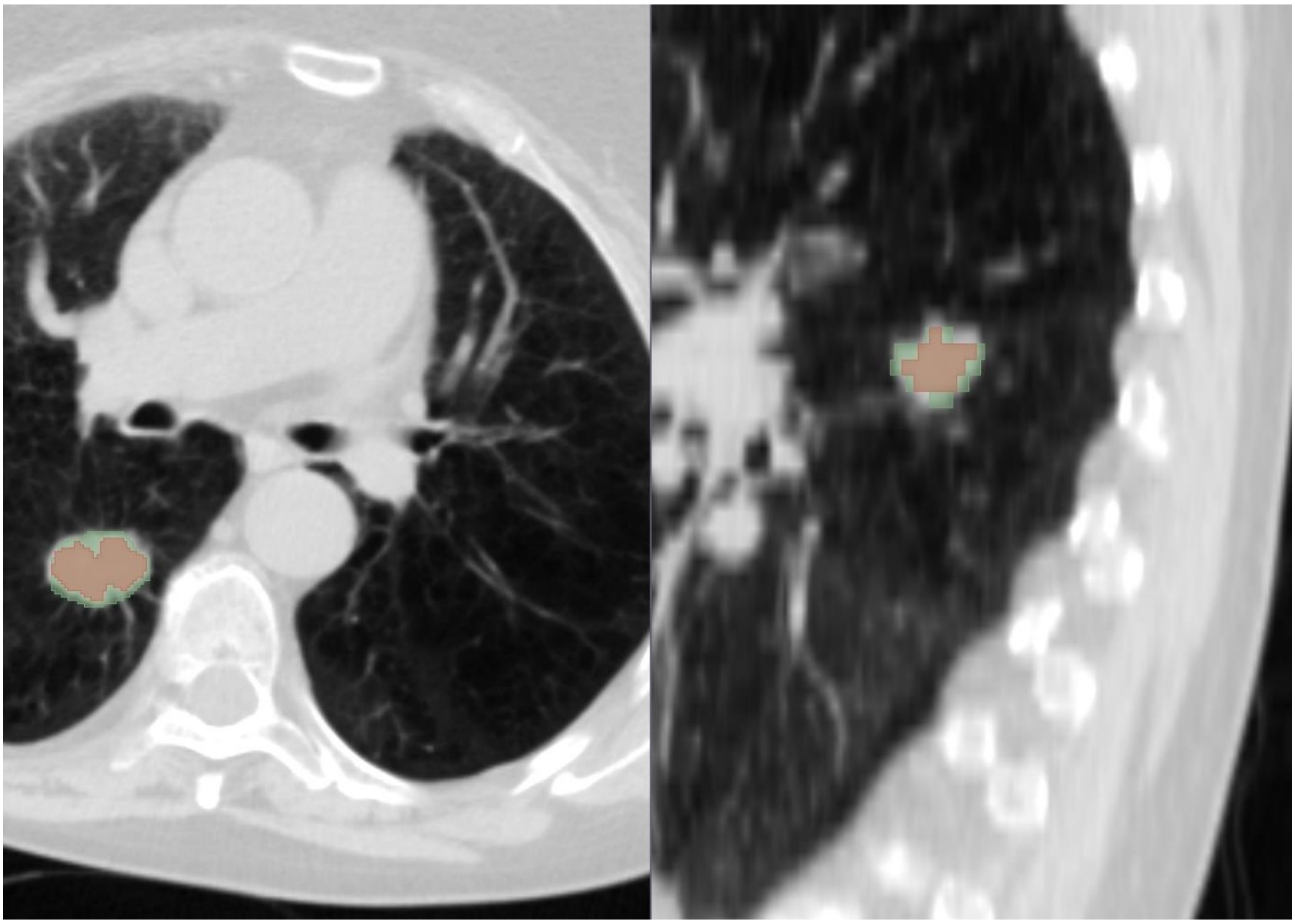

Supplement: Supplementary file 1 — Supplementary file1 (PDF 1083 KB) [file 330_2023_9500_MOESM1_ESM.pdf]
